# A Dermoid Cyst of the Head, Neck, and Face Region: A Case Report

**DOI:** 10.7759/cureus.52152

**Published:** 2024-01-12

**Authors:** Padmashri P Kalmegh, Swati K Patil, Alka Hande, Archana M Sonone, Sakshi Akolkar, Aayushi Pakhale

**Affiliations:** 1 Oral and Maxillofacial Pathology, Sharad Pawar Dental College and Hospital, Datta Meghe Institute of Higher Education and Research, Wardha, IND; 2 Oral Pathology and Microbiology, Sharad Pawar Dental College and Hospital, Datta Meghe Institute of Higher Education and Research, Wardha, IND

**Keywords:** malignant transformation, congenital dermoid inclusion, benign neoplasm, midface abnormalities, dermoid cyst

## Abstract

Dermoid cyst (DC), a rare benign tumor of developmental origin that develops from mesoderm and ectoderm, is frequently identified in children. DC refers to three cysts that are histologically related, namely, DC, epidermoid cyst, and teratoma. About 70% of DCs are discovered in children aged five years or younger, with the majority being congenital. DC of the head and neck are rare, accounting for only 7% of all such cysts. DC, or benign cutaneous tumors, tend to grow and persist. The presence of epithelial cells along the lines of embryonic closure results in a DC. It is always difficult to properly diagnose these lesions using clinical tests and conventional radiography. Histologically, a DC must have two germ cell layers, and the diagnosis can only be made with pathologic confirmation. Specialized imaging tests including CT, MRI, ultrasonography, and histological examinations should be performed to make a diagnosis and choose the best course of action for surgery.

## Introduction

A variety of congenital midface abnormalities can occur in children as a result of improper embryologic development [1]. Dermoid cysts (DCs) account for 1% to 2% of whole-body dermoids and 11% to 12% of head and neck (H & N) dermoids. Several locations, such as the forehead, anterior fontanelle, orbital and periorbital regions, tongue, neck, and nose, can develop facial DCs [2]. Soft tissue abscesses with fistulas can recur due to embryonic epithelial remnants [3]. A typical characteristic of DC is constant growth with occasional episodes of severe inflammation. Pathogenesis includes partial neuroectoderm obliteration in the developing frononasal area [4]. On the wall, mature skin appendages may be present, while keratin and hair may be present in the lumen [5]. DCs have cutaneous appendages such as hair follicles, sweat glands, and sebaceous glands and are surrounded by keratinizing squamous cells [6]. Imaging examinations are required to confirm intracranial and intraorbital extension. Histopathological confirmation is required to confirm the diagnosis [7]. DC is classified as exophytic or endophytic, superficial or deep. Deep DCs are detected based on their location and the continuity of the cyst wall [4]. The severity and location of the lesion affect the surgical strategy, which might range from local excision to an intracranial-extracranial approach [8]. Serious neurological problems can result from DC with intracranial extension [6]. For these cystic entities, early detection and intervention are crucial [9].

## Case presentation

**Figure 1:**
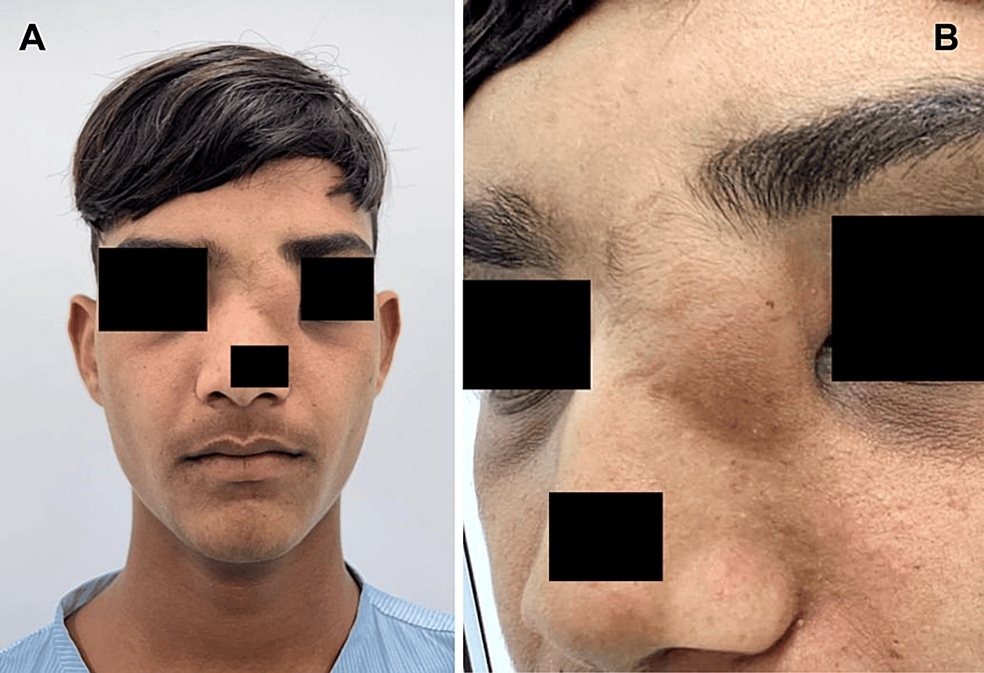
Extraoral swelling.

A 17-year-old male patient complained of swelling over the face for 15 years. The patient had no issues at the time of birth and two years after birth. After that, he started noticing swelling at the junction of the forehead and nose on the left side. Initially, it measured 0.8 x 1 cm in size and increased to a current size of 3 x 2.5 cm approximately (Figure 1A). On extraoral examination, a superoinferior lesion extended from the region of nasion to the infraorbital ridge over the left side and anteroposteriorly extended from the midline of the nose to 1.5 cm short of the columella to the line parallel to the lateral surface of the nose (Figure 1B). The swelling was roughly oval in shape and firm, non-compressible, and non-tender with no rise in local temperature. The transillumination test was negative. No other skin lesions were found elsewhere on the body. The examination of the hair, nails, and mucous membranes was normal. No intraoral extension of the growth was evident. No lymph nodes were palpable. The patient did not provide a history of trauma, nasal discharge, or headache. There was no telangiectasia or pitting on the skin. The physical examination showed a correctly shaped nose, an unbiased septum, a small double inferior turbinate, and an unobstructed nasal cavity on both sides.

**Figure 2:**
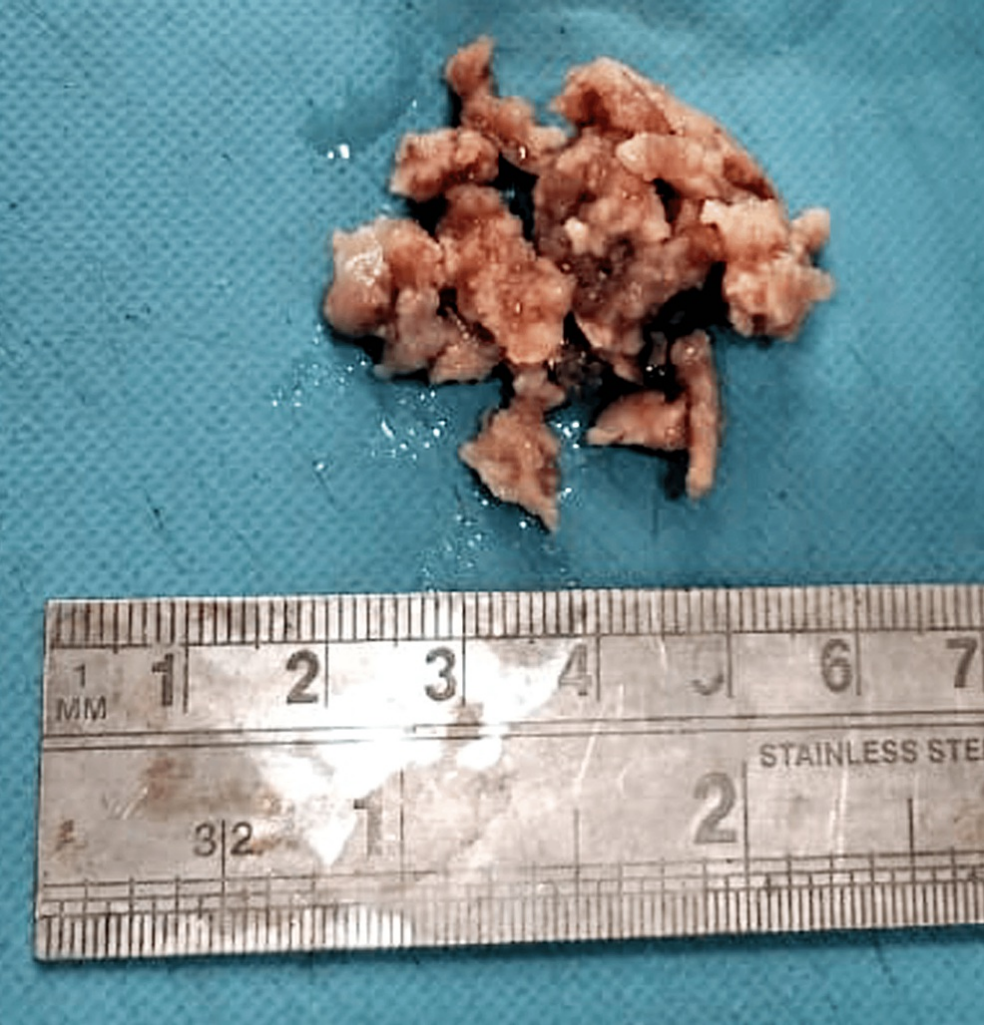
Gross specimen.

Macroscopically, we noted multiple, irregular, brownish, soft specimens measuring 2 x 1.8 x 2.5 cm in size (Figure 2).

**Figure 3:**
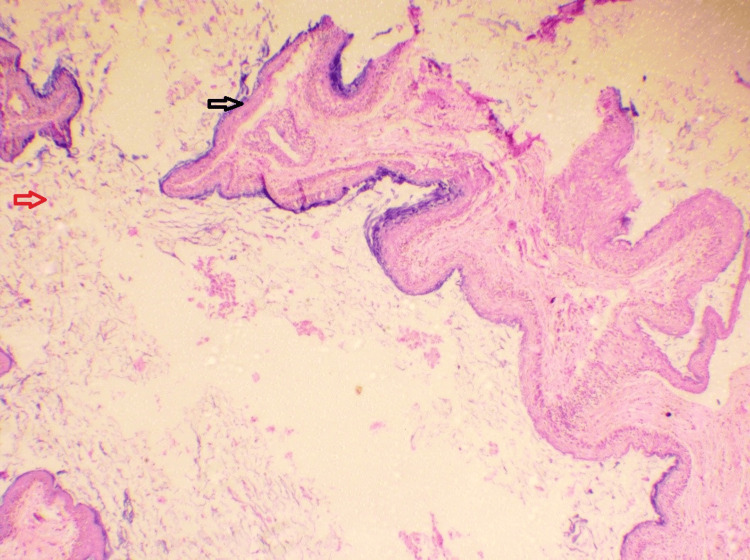
Microscopic presentation: hematoxylin and eosin-stained section under low-power view (10×).

Microscopically, under low-power view, the hematoxylin and eosin (H&E)-stained tissue section showed a cystic cavity (Figure 3, black arrow) and surrounding connective tissue wall (Figure 3, red arrow).

**Figure 4:**
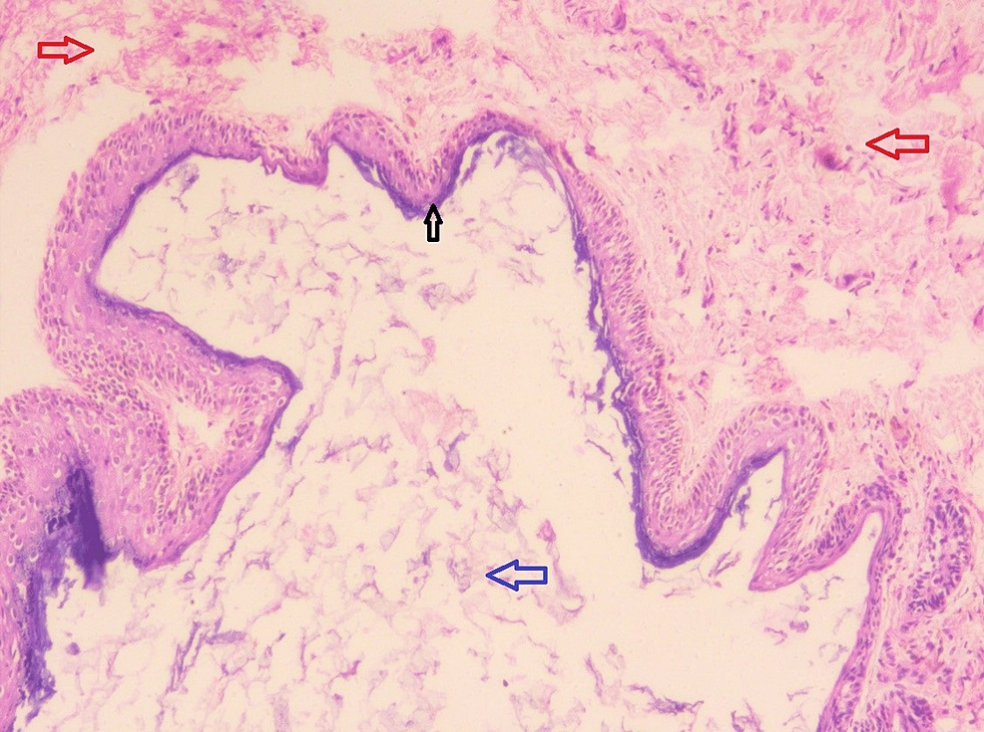
Microscopic presentation: hematoxylin and eosin-stained section under low-power view (40×).

Under a high-power view, the H&E-stained section showed a cystic cavity lined by ortho-keratinized stratified squamous epithelium of varying thickness (Figure 4, black arrow) and connective tissue wall (Figure 4, red arrow). The presence of abundant keratin in the cystic lumen was noted (Figure 4, blue arrow). The connective tissue wall showed skin adnexal structures such as sebaceous glands and hair follicles. The collagen fibers were densely packed. Inflammatory cell infiltrates were chiefly composed of lymphocytes.

## Discussion

DC, also known as benign congenital tumors, form in the early stages of infancy in ectodermal and mesodermal tissues that are inadequately separated because of the merging of the tissues to the suture of the line bone. These tumors are known to be formed from pluripotent cells [6]. They can be discovered as early as infancy, although the age of the patient at manifestation varies widely. Sudden fluctuations in size may make identifying them more challenging. The H & N region contains more than 80% of DCs or roughly 7% of all cysts. The most often reported areas are the periorbital, nasal, submental, and suprasternal areas. Because these neoplasms can appear in a variety of sites, patients may visit other specialists. All of these professionals need to be knowledgeable about the various methods of assessment and therapies that are available [7]. The incidence is predicted to be 1:20,000 to 1:40,000 births [10,11]. Although a DC can occur anywhere beneath the skin, 80% are located in the H & N region, primarily in the orbital and periorbital regions, particularly superotemporally close to the fronto-zygomatic suture [3]. The majority of reported cases include the ovaries, testicles, and midline of the body. There is no sex preference. They occur as gradually expanding masses in the second or third decade of life [12]. They are infrequently hereditary and are linked with other malformations [13]. DCs appear as a tiny pit, a fistula significantly approaching the nasal tissues, or a uni- or multi-loculated lesion in the center of the nose. Cystic lesions are often hard, non-pulsating, non-transilluminating, non-compressible, and painless nodules [1].

DC typically presents as a firm, non-compressible, non-pulsatile mass or a lesion on the midline of the nose that resembles a furuncle [14]. They have been categorized into superficial and deep cysts based on proximity to the inter-zygomztic line on a CT scan, as well as exophytic and endophytic cysts based on their approach to the orbital rim. Usually, superficial DCs are detected early, although deep tumors often appear later. On the other hand, DCs deep in the orbit frequently present as a painless, gradual-onset proptosis in young adults [4]. Parents generally detect a painless lump or swelling along the orbital rim during the first year of life, which is indicative of periorbital or superficial cysts. They are confined to the area above the nasal bridge or at or near the glabella [15]. Nonetheless, a tiny subset of lesions is currently established to constitute the tip of the iceberg, i.e., a superficial manifestation of a more profound, widespread pathogenic phenomenon. DC and sinuses have traditionally been categorized into the following three pathologic types: (i) acquired, or the implantation type; (ii) congenital, of the teratoma type; and (iii) congenital, of the inclusion type. The congenital orbitofacial DC are typical of the inclusion variety and rarely manifest as teratomas. These aggregates are believed to result from dermal and epidermal cells being displaced into and along embryonic lines of fusion. Subsequently, these cells develop into solid and cystic structures, which may or may not have deep and superficial sinus pathways [16]. It is believed that DCs of the H & N region belong to the congenital inclusion category [7].

One etiology is the partial destruction of neuroectoderm in the developing frontonasal region [10,17]. A brain abscess, meningitis, soft tissue and skeletal deformities, and local infection can result from a progressive expansion of nasal DC. The only available treatment option is surgical excision, and prompt diagnosis is crucial. During embryogenesis, the nose forms out of three layers, namely, ectoderm, mesoderm, and a deeper layer of the cartilaginous capsule. The nasal and frontal bones grow through intramembranous ossification in the mesoderm during the eighth and ninth weeks of pregnancy but are divided by the fonticulus nasofrontalis [18]. During this time, the nasal bone and the deeper cartilaginous capsule form the prenasal gap. The dura has a little protrusion that makes contact with the skin. The dura’s protrusion is surrounded by the foramen cecum, and the skin separates from it when the frontal bone’s nasal process expands. Normally, the dura obliterates, obliterating the neuroectodermal connection. The cribriform plates form and the fonticulus nasofrontalis and foramen cecum unite when the neuroectodermal link is destroyed. The pathophysiology of DC is well characterized in the literature. In 1893, Bland-Sutton proposed the superficial sequestration theory [19]. The embryonic growth of the medial nasal processes begins to combine during the fourth or sixth week. According to this view, a cyst or a sinus might form as a result of epithelial trapping during the union of medial nasal processes. This does not explain the establishment of a dermoid with cerebral extension, but it does explain the formation of superficial nasal DC [19,20]. Littlewood proposed the trilaminar theory in 1961. This theory suggests that by the second month of gestation, nasal cartilaginous tissue has fully formed. The septum is composed of a fine epidermal layer surrounded by two cartilaginous layers. The middle ectodermal layer breaks down in the third month of pregnancy, and it is thought that a sinus or cyst occurs as a result of the dural-derived ectoderm remaining [10]. Grunwald’s 1910 proposal, later referred to as the prenasal theory by Pratt and the cranial theory by Bradley, is the most widely recognized hypothesis. The theory is based on the observation that dermal attachments might follow the course of the neuroectodermal tract as it recedes. A sinus or a cyst may occur as a result of the dura mater pulling the nasal epiderm upward and inward when it recedes from the prenasal region. Depending on how it connects to the nasal dorsal skin, the ensuing epithelial lining forms a DC [10,21].

Three histological types are visible in DC, namely, teratoma, epidermoid cyst, and DC. Epidermoid cyst lacks adnexal parts, a simple squamous epithelium with a fibrous wall surrounding it. True DC is an epithelial-lined cavity that has sweat and sebum glands as well as hair follicles in the cyst wall, along with keratinization. A compound cyst, also known as a teratoid cyst, is lined by ciliated to simple squamous epithelia and contains derivatives of ectoderm, mesoderm, and endoderm. Each of the three groups can have some keratinous content [9]. DC and sinuses histologically replicate the characteristics of normal skin. They consist of a keratinous material-filled cavity walled with stratified squamous epithelium that includes fibroadipose tissue, hair follicles, sebaceous glands, and, less frequently, sweat glands [22]. In addition to nasal gliomas, nasal encephalocele, congenital hemangiomas, and epidermoid cysts, meningocele or meningoencephalocele, cystic schwannoma, hydatid cyst, epithelial inclusion cyst, and cold abscess are among the other differential diagnosis for DC [15,23]. Otolaryngologists have been concerned about the difficulty of differentiating between a duplication abnormality of the first branchial cleft or pouch and a true DC of the temporal bone. Because of the possible close association between a duplication abnormality and the route of seventh cranial nerve, this structure may be in danger during dissection. The preoperative conversation and surgical plan are significantly changed by the probability of cranial nerve VII involvement. There does not seem to be any discernible microscopic distinction in the development of a DC and duplication anomalies of the first branchial cleft, according to the histologic review of these temporal bone disorders [7]. It is uncommon for a DC to undergo malignant transformation. The most frequent change is to squamous cell carcinoma. When patients initially report, they are often in an advanced stage, with a very bad prognosis [24]. Excision without damaging the cyst wall is the accepted treatment of choice for managing DCs. In the presented case, the lesion was managed by excision without damaging the cyst wall. The lesion was simply removed with surgical excision, with no resistance to traction. Then, a deep local curettage and a 2-0 silk suture were performed. The differential diagnoses in our patient included nasal glioma, encephalocele, congenital haemangioma, sebaceous cyst, lipoma inflammatory or infectious lesions, facial trauma sequelae, benign neoplasms, and congenital or developmental masses, with epidermoid cyst. A small, asymptomatic DC can be stable for years or even regress, in which case it might not need to be removed right away. Still, the majority of cysts continue to grow slowly. To prevent potential problems, excision should be done as soon as the cyst is discovered [5]. Our patient was followed up for three months.

In general, DCs grow slowly. It is observable if there is no continual increase. If not, it needs to be removed immediately and submitted for a histological analysis. The best course of action is total excision of the cyst wall. Surgery might not be able to remove the cyst wall entirely if it is tightly related to any significant structures, which could result in a recurrence. Recurrence can result in cerebral infection, abscess formation, and possibly even death [25]. If DCs have a cerebral extension, a nasal median incision with a nasal bone osteotomy, pericranial flap, and keyhole-type craniotomy is recommended [26]. The majority of DCs merely have skin scars on their body, with favorable prognoses. A favorable outcome alongside a low incidence of recurrence is connected with a multidisciplinary collaborative approach and surgery for individuals with intracranial extension. After a six-month period of recovery, the nasal tip healed entirely with no visible scarring and the septum of the nose area was repaired thoroughly. Moreover, the local mucosa was unimpeded without recurrence. However, an extended period of follow-up is required [27].

## Conclusions

The complex anatomic locations in which DC originates give rise to a variety of lesions with a wide range of localizations and characteristics. It is mainly caused by entrapped ectoderm at embryologic bony fusion sites. Congenital DC in the orofacial area is a heterogeneous group of lesions for which the only known effective therapy is excision. DCs are removed to avoid infection, establish a histopathological assessment, and improve an aesthetically deforming condition. Recurrence is rare if complete excision of the lesion is performed.
